# Microrheology of *Pseudomonas aeruginosa* biofilms grown in wound beds

**DOI:** 10.1038/s41522-022-00311-1

**Published:** 2022-06-15

**Authors:** Minhaz Ur Rahman, Derek F. Fleming, Liyun Wang, Kendra P. Rumbaugh, Vernita D. Gordon, Gordon F. Christopher

**Affiliations:** 1 Department of Mechanical Engineering, Whitacre College of Engineering, Texas Tech University, Lubbock, TX USA; 2 Department of Surgery, Texas Tech University Health Sciences, Lubbock, TX USA; 3 Department of Physics, Center for Nonlinear Dynamics, Interdisciplinary Life Sciences Graduate Programs, LaMontagne Center for Infectious Disease, The University of Texas at Austin, Austin, TX USA

**Keywords:** Biofilms, Biological techniques

## Abstract

A new technique was used to measure the viscoelasticity of in vivo *Pseudomonas aeruginosa* biofilms. This was done through ex vivo microrheology measurements of in vivo biofilms excised from mouse wound beds. To our knowledge, this is the first time that the mechanics of in vivo biofilms have been measured. In vivo results are then compared to typical in vitro measurements. Biofilms grown in vivo are more relatively elastic than those grown in a wound-like medium in vitro but exhibited similar compliance. Using various genetically mutated *P. aeruginosa* strains, it is observed that the contributions of the exopolysaccharides Pel, Psl, and alginate to biofilm viscoelasticity were different for the biofilms grown in vitro and in vivo. In vitro experiments with collagen containing medium suggest this likely arises from the incorporation of host material, most notably collagen, into the matrix of the biofilm when it is grown in vivo. Taken together with earlier studies that examined the in vitro effects of collagen on mechanical properties, we conclude that collagen may, in some cases, be the dominant contributor to biofilm viscoelasticity in vivo.

## Introduction

Bacterial biofilms are three-dimensional, viscoelastic structures composed of metabolically diverse bacteria aggregated together by a matrix of hydrated extracellular polymeric substances (EPS)^1^. Among bacterial biofilm formers, *Pseudomonas aeruginosa* is one of the most harmful opportunistic pathogens, often capable of withstanding the host immune system response and antimicrobial agents^2,3^. Persistent *P. aeruginosa* biofilms infect immunocompromised lungs, chronic wounds, and burns^4–6^. On average, 6 million patients are reported with *P. aeruginosa* infected wounds every year in the USA alone^7–9^, and worldwide costs of these infections exceed billions of dollars^10,11^. Patients face prolonged treatments^12^, which can include mechanical debridement of necrotic tissue in hopes of removing the biofilm and/or increasing its susceptibility to treatment. Unfortunately, debridement often must be repeated frequently, is a painful, time intensive process, and may not effectively remove the biofilm^13–16^.

The efficacy of debridement depends in part on understanding how interaction between bacteria-produced EPS and materials originating in the host environment alter biofilms’ mechanical properties. However, most studies of mechanics use in vitro grown biofilms^17,18^, and even the best efforts at mimicking in vivo conditions cannot replicate the complex host environment and its interaction with EPS^19,20^. Unfortunately, it is technically very difficult to design methods capable of characterizing the mechanics of biofilms in vivo. These technical issues prevent clear understanding of how the host environment affects biofilm viscoelasticity.

We have developed a new methodology to characterize in vivo biofilms using ex vivo microrheology. In this technique, *P. aeruginosa* biofilms are grown in vivo in wound beds of a mouse model of chronic wound infection^21–23^. During growth, fluorescent microparticles are embedded into the biofilm, allowing particle-tracking microrheology to characterize viscoelasticity^24^. Microrheology measurements are done ex vivo on biofilms excised from the wound beds as soon as possible after excision. Therefore, the ex vivo measurements characterize the mechanical properties reflective of in vivo conditions. To the best of the authors’ knowledge, such ex vivo studies have not been previously reported.

Ex vivo results were then compared to those measured during in vitro experiments^25^. Biofilms exhibited different mechanical properties ex vivo than in vitro, suggesting that interactions with the host environment change the mechanical properties of *P. aeruginosa* biofilms. This is the first time this has been shown for any type of biofilm or infection site. In the future, the innovative methodology used in this study can be extended both to other species of biofilm formers and to other sites of infection. Thus, we demonstrate a new tool that opens the possibility of ex vivo investigations of medically important biofilms, grown in vivo, for which there previously was no extant method.

## Results

### Effectiveness of ex vivo microrheology

**Fig. 1:**
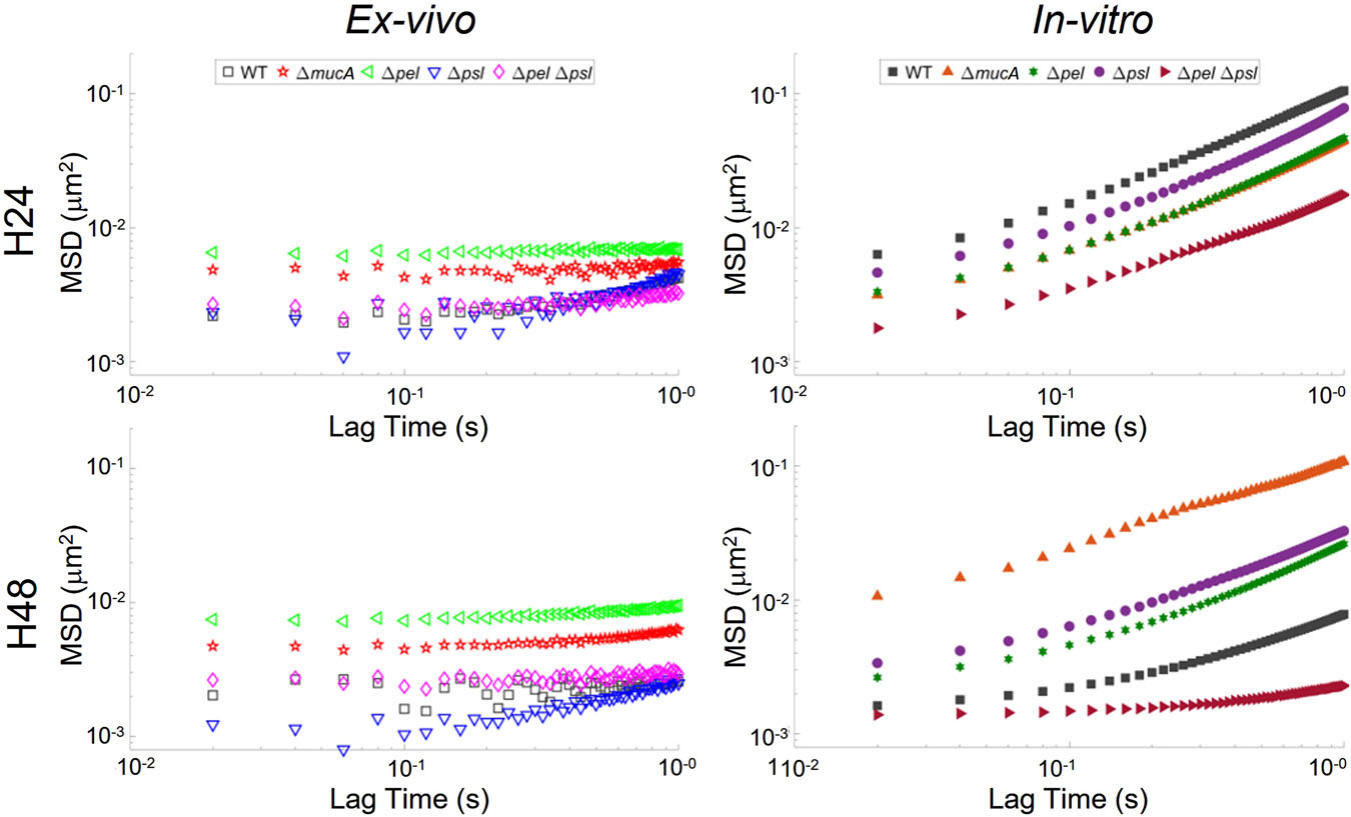
Ensemble averaged MSD-lag time plots from ex vivo and in vitro experiments at 24 and 48 h. In vitro data reproduced from ref. ^25^ with permission from the Royal Society of Chemistry.

The ensemble ex vivo MSD curves (Fig. 1) are linear on a log-log plot, indicating that the data reflect thermal motion of particles^24^ and can be analyzed using traditional microrheology methods. The results clearly show the new methodology used can produce quantitative results, making the technique a viable alternative to in vitro studies moving forward. However, the ex vivo data does appear noisier than the in vitro curves (Fig. 1). There are several possible factors underlying this increased noise.

The first possible consideration is possible biological reasons for increased variability/noise in the ex vivo results. In vitro experiments have environments that are highly controlled - microchannels that have identical surfaces, growth medium, and temperature; even with such control, we know that there is a great deal of variability in biofilm rheological results^26^. However, the ex vivo biofilms were grown in mouse wounds that contain varying levels of pH, oxygen, small molecules, collagen, fibrin, elastin, fibroblasts, neutrophils, blood vessels, immune cells, enzymes, and more which are heterogeneous throughout the wound and particular to each specimen^27^. Determining how concentrations of these materials change within and between samples was beyond the scope of this work. However, these changes to growth conditions could affect viscoelasticity and create more “noise” or variation in data. If this were the case, we would expect to see a greater heterogeneity in ex vivo results when looking at individual tracks of data.

**Fig. 2:**
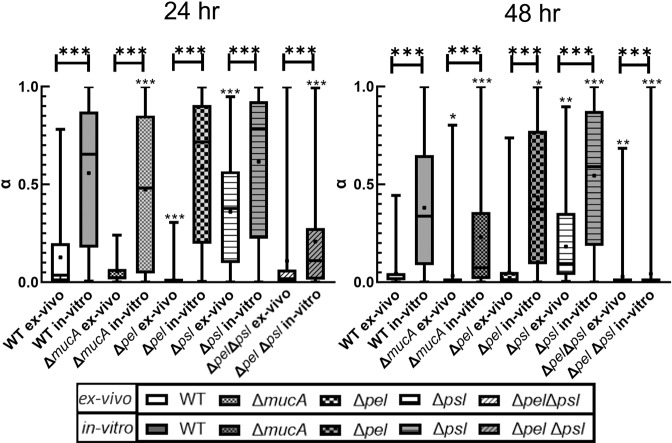
Box-whisker plot with statistical comparison of α-values of individual trajectories for particles. A box represents the middle 50% range of data, lines within boxes are the median of the distribution, the black dot represents the mean, and the upper/lower vertical line represents the upper/lower quartiles. Star values directly over each strain indicate comparison to the respective WT PAO1 (ex vivo for ex vivo, and in vitro for in vitro) done using a non-parametric Kruskal–Wallis one-way ANOVA method with Dunn’s multiple comparisons among tested groups. Stars values over horizontal lines compare a strain’s ex vivo and in vitro measurements done using a Mann–Whitney test. *P* ≤ 0.05 demarcated with *, *P* ≤ 0.01 demarcated with **, and *P* ≤ 0.001 demarcated with ***. *P* ≤ 0.05 demarcated with *, *P* ≤ 0.01 demarcated with **, and *P* ≤ 0.001 demarcated with ***. In vitro data reproduced from ref. ^25^ with permission from the Royal Society of Chemistry.

Analysis of the distributions of α from individual particle tracks can reveal mechanical heterogeneity to determine whether observed noise in ex vivo data comes from biological variability. Examining each bacterial strain, the 50% boxes in Fig. 2 are smaller for the ex vivo biofilms than for the in vitro biofilms. Typically, the whiskers of the ex vivo distribution are also smaller than those for the same strain’s in vitro distribution. Therefore, the ex vivo biofilms are more homogenous than in vitro, indicating that biological variability between the two environments is comparable or smaller in the ex vivo environment. Therefore, the noise in Fig. 1 derives from other technical aspects of the methodology.

It is possible that ex vivo imaging conditions also affected particle tracking resolution. Although all in vitro and ex vivo data were recorded using the same camera, different microscopes and objectives were used as described in the Methods section. This resulted in slightly different spatial resolution in the digital images acquired for the two types of experiment. However, all spatial resolutions were above the minimum standards needed for accurate particle tracking. When doing our analysis, we did not experience differences in the difficulty in tracking in-focus particles between ex vivo and in vitro experiments.

The main reason for increased noise is that fewer particle tracks are acquired for the ex vivo biofilms than for the in vitro biofilms (Table 4). Because of the need to maintain live animals, it was costly and time intensive to prepare in vivo biofilms for visualization. Therefore, for this proof-of-concept study, we used a limited number of animals, which limited the total number of ex vivo experiments. Consequently, the ex vivo ensemble curves are built from fewer individual particle tracks than are the in vitro ensemble curves, thus reducing statistical robustness and generating a noisier result. However, this problem can be rectified by using more biological replicates in future studies.

Additionally, methodological differences affect the number of particle tracks obtained, per replicate, in each experiment type. It appears there is less embedment of particles during in vivo biofilm growth than during in vitro growth, resulting in fewer total particles embedded in ex vivo biofilms. This is likely due to differences in biomass between the two growth conditions: in vivo biofilms are smaller in size whereas in vitro biofilms can be larger and limited only by channel space. Furthermore, optical differences affected the number of particle tracks acquired. The transparent microfluidic channels used for in vitro measurements contain only the biofilm and particles; many particles and large areas of biofilm can be observed in the volume defined by the field of view and the depth of field, and the regions containing the biofilm are well-delineated. The excised tissue and biofilm used in ex vivo studies are optically denser and more variable, and have higher levels of ambient fluorescence, than in vitro samples. Furthermore, in ex vivo samples, particles were embedded in tissue and biofilm outside the focal plane imaged for particle tracking. This made it more difficult to distinguish particles within the biofilm from particles in the tissue surrounding the biofilm, requiring exclusion of a higher percentage of particles from tracking analysis. The overall result of these combined effects was that ex vivo experiments averaged 34.4 particle tracks per technical replicate whereas in vitro experiments averaged 54.1.

Overall, the new methodology can be used for ex vivo microrheology, and perceived issues in noise can be corrected by using larger sample sizes to improve statistics and/or adjusting microscopy setups to improve imaging.

### Comparison of ex vivo to in vitro data relative viscoelasticity

Table 1 shows α values extracted from the ensemble data in Fig. 1. For in vitro biofilms, WT biofilm became more relatively elastic from 24 to 48 h. In vitro, the Δ*mucA* biofilm had α values identical to those of WT at 24 h but this value did not change with age. It is, therefore, less relatively elastic than the WT at 48 h. This appears to agree with previous findings that increased alginate production results in biofilms that have lower yield moduli both in vitro^28^ and in ex vivo samples that were homogenized using mechanical agitation^29^. After 24 h, in vitro *∆pel* biofilms were slightly more elastic than WT biofilms, although there was substantial overlap in the confidence bounds. After 48 h, the *∆pel* biofilm became more relatively elastic but was much less elastic than the WT biofilm at 48 h. In previous in vitro studies, the lack of Pel was found to make biofilms more elastic, as was observed here at 24 h^28,30^. In vitro *∆psl* biofilms also became more relatively elastic from 24 to 48 h. However, the 48 h *∆psl* biofilm was less relatively elastic than the 48 h WT biofilm. This is consistent with previous in vitro work that found the presence of Psl resulted in more elastic biofilms^28,30^. The *∆pel∆psl* biofilms, whose matrices are composed of proteins, eDNA, and some alginate, are more relatively elastic than the WT at both time points.

**Table 1:** Mean ensemble α of MSD-Lag time curves extracted from Fig. 4 with lower and upper bounds based on a 95% confidence interval.

	In vitro				
	WT	Δ*mucA*	Δ*pel*	Δ*psl*	Δ*pel* Δ*psl*
24 h	0.57 (0.52, 0.72)	0.52 (0.44, 0.61)	0.49 (0.39, 0.60)	0.54 (0.47, 0.61)	0.40 (0.27, 0.52)
48 h	0.18 (0.11, 0.25)	0.56 (0.45, 0.67)	0.38 (0.30, 0.47)	0.43 (0.35, 0.51)	0.04 (0.02, 0.05)
	Ex vivo (Mouse)				
24 h	0.27 (0.23, 0.31)	0.05 (0.02, 0.07)	0.03 (0.02, 0.04)	0.36 (0.30, 0.43)	0.09 (0.07, 0.11)
48 h	0.04 (0.0, 0.08)	0.10 (0.09, 0.12)	0.08 (0.07, 0.09)	0.34 (0.30, 0.38)	0.05 (0.03, 0.07)

In vitro data reproduced from ref. ^25^ with permission from the Royal Society of Chemistry.

In summary, the results of these in vitro studies are generally consistent with previously published in vitro studies. Differences between this data and published results likely derive from differences in rheological and culturing methods, which we have discussed in detail elsewhere^26^.

When comparing ex vivo to in vitro results some similarities were observed. The ex vivo α values showed that the WT biofilm in vivo stiffens over time, as does the in vitro (Table 1). The ex vivo *Δpsl* biofilms also behaved over time like in vitro biofilms. Based on the confidence bounds of *α*, at 24 h both in vitro and ex vivo *Δpsl* biofilms had similar relative elasticity to their respective WT, and after 48 h neither film had become more relatively elastic. The latter is shown by the overlap of the 95% confidence bounds of the respective 48 h and 24 h *α*. Finally, after 48 h, almost all exopolysaccharide mutant strains were statistically different from their corresponding WT except the ex vivo Δ*pel* (Fig. 2).

However, there were clear differences between the ex vivo and the in vitro biofilms. All ex vivo biofilms consistently had lower values of α than the corresponding in vitro biofilms (Table 1). This indicates that the ex vivo biofilms were more relatively elastic. These observations are quantitatively confirmed through statistical analysis. Regardless of EPS production, at both 24 and 48 h, the means/medians of the α distributions (Fig. 2) show that all ex vivo biofilms are more relatively elastic than the corresponding collagen-free in vitro biofilms with high statistical significance (indicated with ***). Therefore, we confirm statistically that the biofilms grown in vivo are different from, and more relatively elastic, than their in vitro, collagen-free counterparts.

There were also differences in aging behavior. Each ex vivo strains’ *α* values, excluding WT, were within 95% confidence bounds of each other at 24 and 48 h (Table 1). This indicates less effect of aging on relative elasticity. In vitro, only *ΔmucA* and *Δpsl* biofilms showed similar behavior.

Finally, we see differences in the behavior of individual strains between the ex vivo and in vitro experiments. In vitro, Δ*mucA* and Δ*pel*Δ*psl* were both statistically different from the WT, whereas the Δ*pel* and Δ*psl* were statistically the same as the WT (Fig. 2); ex vivo results are the exact opposite. After 24 h, ex vivo *ΔpelΔpsl* and *ΔmucA* biofilms were more relatively elastic than the ex vivo WT unlike the in vitro results (Table 1). At 24 h, e*x vivo Δpel* biofilms were more relatively elastic than the corresponding WT biofilms unlike in vitro results (Table 1); this ex vivo behavior appears consistent with previous published results^28,30^. In general, the differences in various strains in comparison to WT for both conditions indicate that the in vivo roles of specific exopolysaccharides are not the same as has been observed in vitro when it comes to biofilm mechanical properties.

The reasons that *α* is different for in vivo and in vitro biofilms grown by the same bacterial strain, and that changes in α with varying exopolysaccharide and age are also different for in vivo and in vitro biofilms are not discernable alone by these tests. These effects may be related to greater metabolic activity and biomass accumulation that has been observed for ex vivo *P. aeruginosa* biofilms in comparison to in vitro^31^. Differences in attachment to tissue surfaces and PDMS may affect biofilm microstructure or expression of particular EPS components. Additionally, differences in host temperature and pH from in vitro conditions might alter biofilm mechanical properties. Finally, changes in both cell density and film thickness, which were not possible to measure with the microrheology setup, may also impact viscoelasticity^32,33^.

Our previous study suggested that host-derived extracellular matrix (ECM) components at wound sites can create a dynamic microenvironment that affects biofilm growth^34^. In particular, collagen in host ECM may affect the surface attachment and accumulation of biomass of *P. aeruginosa* biofilms^35^. Our own recent rheological investigation demonstrated that growth in the presence of collagen, a major ECM component, increased the relative elasticity of *P. aeruginosa* biofilms^25^. In that study, *P. aeruginosa* biofilms were grown in vitro in a wound-like medium containing 20% collagen^25^. For each strain tested, median values of in vitro α in the presence of collagen were dramatically lower than values for in vitro with no collagen. In that paper, we suggested that the collagen incorporated into the biofilm and became a de facto component of the EPS through physical entanglement^25^. Our previously-published results for biofilms grown in vitro in the presence of 20% collagen^25^ (Table 2) are similar to our new ex vivo results (Table 1). Compared with the collagen-free in vitro biofilms, both ex vivo biofilms and biofilms grown in vitro with collagen have lower relative elasticity and demonstrate less change from 24 to 48 h. The ex vivo Δ*psl* biofilm was substantially more viscous than the in vitro collagenated biofilm, but both conditions showed little to no aging. Furthermore, the ex vivo *results* and the in vitro with additional collagen are more homogenous than in vitro without collagen (Table 2)^25^. Therefore, we infer that the shifts in biofilm mechanics associated with in vivo growth, compared with collagen free in vitro growth (i.e., greater relative elasticity and less aging), likely arise in large part from the incorporation of collagen from the wound bed into the biofilm EPS. If true, this may mean that host collagen is dominant, or at least a primary, matrix component for biofilm infections in wounds.

**Table 2:** α-fitted (reprinted from recent study conducted by Rahman et al.^25^ Collagenated Wound model (20% free collagen in vitro model).

	In vitro 20% collagen				
	WT	Δ*mucA*	Δ*pel*	Δ*psl*	Δ*pel* Δ*psl*
24 h	0.11 (0.04, 0.17)	0.09 (0.07, 0.1)	0.08 (0.06, 0.10)	0.06 (0.05, 0.07)	0.18 (0.15, 0.21)
48 h	0.06 (0.05, 0.07)	0.10 (0.08, 0.12)	0.11 (0.08, 0.14)	0.08 (0.06, 0.10)	0.08 (0.07, 0.09)

Data reproduced from ref. ^25^ with permission from the Royal Society of Chemistry.

### Zero time creep compliance analysis

**Fig. 3:**
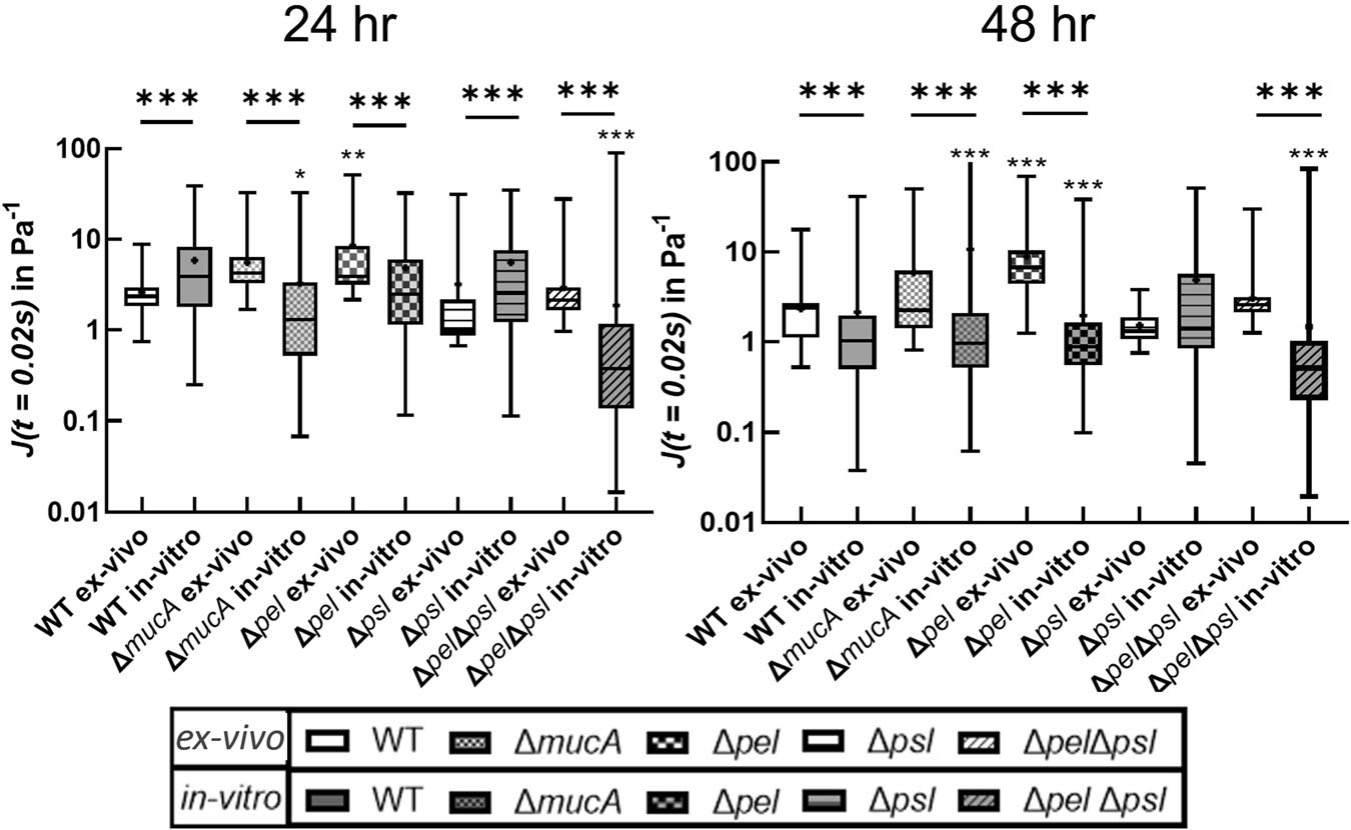
Box whisker plot at semi-log scale showing the creep compliance measured using individual tracked particles. A box represents the middle 50% range of data, lines within boxes are the median of the distribution, the black dot represents the mean, and the upper/lower vertical line represents the upper/lower quartiles. Star values directly over each strain indicate comparison to the respective WT PAO1 (ex vivo for ex vivo, and in vitro for in vitro) done using a non-parametric Kruskal–Wallis one-way ANOVA method with Dunn’s multiple comparisons among tested groups. Stars values over horizontal lines compare a strain’s ex vivo and in vitro measurements done using a Mann–Whitney test. *P* ≤ 0.05 demarcated with *, *P* ≤ 0.01 demarcated with **, and *P* ≤ 0.001 demarcated with ***. *P* ≤ 0.05 demarcated with *, *P* ≤ 0.01 demarcated with **, and *P* ≤ 0.001 demarcated with ***. In vitro data reproduced from ref. ^25^ with permission from the Royal Society of Chemistry.

After both 24 and 48 h of growth, we analyzed the for particle-tracking data for both ex vivo and in vitro biofilms to determine the creep compliance at t = 0.2 s (our first recorded data point (Fig. 3). For most of the bacterial strains, both in vivo and in vitro creep compliance values were within the range 0.1~100 Pa^−1^. In general, that for identical strains/conditions, creep compliance slightly decreases or does not change as biofilms age 24 to 48 h. Increased stiffness with time has been observed in previous work^36^.

In vivo compliances were statistically different from the corresponding bacterial strain’s in vitro compliance for almost all cases, with the sole exception of *Δpel* at 48 h. However, we note that the difference between the means/medians of the compliance of the in vitro in comparison to ex vivo results exhibited no consistent behaviors or trends based on exopolysaccharide composition. In general, these results indicate that in vivo growth conditions create biofilms with different mechanical properties than do in vitro growth conditions and that the role of exopolysaccharides on mechanics as understood from in vitro experiments may not correlate with in vivo results.

Ex vivo, at 24 and 48 h, the creep compliance of each biofilm grown from an exopolysaccharide mutant, except *Δpel*, was statistically similar to the ex vivo WT biofilm. This would seem to indicate that growth in vivo results in more consistent biofilm mechanics regardless of bacterial production of polysaccharides, even though differences in polysaccharide production result in significant mechanical differences in vitro. The statistical difference of the *Δpel* biofilm from the WT is an outlier that we cannot explain. At both 24 and 48 h ex vivo, the *Δpel* biofilm was more compliant than the WT, which seems counter to previous published work on the in vitro effects of Pel^28,30^.

In vitro at 24 h, the Δ*mucA* and Δ*pel*Δ*psl* biofilms were both less compliant than the WT biofilm, whereas the Δ*pel* and Δ*psl* biofilms had compliances statistically-indistinguishable from that of the WT biofilm. These results seem at odds with the previous results on the decreased yield stress of alginate overproducers^28^ and the importance of Psl on elasticity^28,30^. Differences in rheological methodologies and measured properties may explain these differences. In vitro at 48 h, all but one of the mutant biofilms had compliances that were statistically different from that of the WT. The exception, once more, was the *Δpel* biofilm.

These findings indicate that, over time, differences in exopolysaccharide composition can result in significantly different mechanical properties for biofilms grown in vitro. This is strikingly unlike the case for biofilms grown in vivo. This again seems to imply that in vivo environment has significant impact on biofilm mechanical properties and that the in vivo growth environment may be more important for biofilm mechanics than the self-secreted exopolysaccharides.

## Discussion

In general, the results of our in vitro studies appear to be consistent with the results of previously published in vitro studies^28–30^. Biofilms typically stiffen and become more elastic with time; the effects of individual exopolysaccharide components are consistent with the literature. Differences between these data and published results likely derive from differences in rheological and culturing methods, which we have examined in detail previously^26^. We further note that biofilms grown in vitro from strains in which either Pel and/or Psl are not produced or alginate is over produced are consistently different from WT biofilms grown in vitro.

However, the results of our new ex vivo microrheology experiments clearly show that the in vivo wound environment substantially affects the viscoelasticity of *P. aeruginosa* biofilms. In both relative elasticity (as measured by *α*) and overall resistance to deformation (as measured by compliance), after both 24 and 48 h of growth, the ex vivo microrheology results were always statistically different from the corresponding in vitro measurements. In general, the ex vivo systems had lower values of *α* (indicating that they are more relatively elastic), showed less variation in mechanics with changes in the production of different exopolysaccharide components, and are less heterogeneous in both compliance and α than the corresponding in vitro biofilms.

Thus, the strongest finding of this study is that much of the previous characterization of the mechanical properties of biofilms grown in vitro may not correspond well to the mechanical properties of biofilms grown in vivo.

The reasons for the differences between biofilms grown in vitro and biofilms grown in vivo could be changes to surface attachment, growth rate, environmental conditions (temperature, pH, etc…), or nutrient availability. However, we believe one primary mechanism is the incorporation of host ECM into the biofilm matrix. In particular, in previous work^25^, we showed that free collagen appeared to integrate into the biofilm and become a de-facto component of the matrix, decreasing the relative elasticity of biofilms and increasing their homogeneity. The results of that study are very similar to the results of the ex vivo experiments conducted here, indicating the changes in ex vivo biofilms are likely due to integration of ECM components into the biofilm matrix.

This likely has important medical implications for biofilm infection and treatment. For example, in a recent study, enzymes (alginate lyase or DNase) applied to collagen-free in vitro biofilms were found to affect biofilm mechanics the most when the enzyme specificity was matched to a dominant matrix component^36^. Treating in vitro biofilms with enzymes that were not specific to a bacteria-produced dominant matrix component had little effect—glycoside hydrolases, in particular, had no effect on biofilm mechanics^36^. However, for biofilms infecting wounds, glycoside hydrolases are known to cause dispersal of bacteria, which can make them more susceptible to antibiotics^21,37–39^. Although it is not known for certain that dispersal is linked to mechanical compromise of the biofilm matrix, this seems highly plausible. In that light, it is worth noting that dispersal by glycoside hydrolases may be effective due to a currently unknown ability to also break up ECM components that are integrated into the biofilm. Hence, the study of these dispersal agents should in the future be carried out in the presence of ECM components like collagen.

It is also worth noting that, in our previous study, the effects of alginate lyase and DNase on the dispersal of bacteria from biofilms grown in vivo was anti-parallel to their effect on the mechanics of biofilms grown in vitro; the bacterial strain with the least enzyme-induced change in in vitro biofilm mechanics had the greatest enzyme-induced dispersal ex vivo^36^. This may constitute another hint that matrix composition, and consequent response to treatment in vivo is substantially different from that in vitro. This implies that changes to biofilm properties caused by the growth environment may have important consequences for disease and treatment. In future, research into biofilm disease and treatment needs to consider more than bacterial-produced components this has already begun in some cases^40^.

## Methods

### Bacterial strains

The EPS is the major component of the biofilm, making up ~90% of dry biofilm mass and is partially responsible for regulating microenvironment and microstructure^41–44^. The EPS of *P. aeruginosa* biofilms contains three polysaccharides, Pel, Psl, and alginate, which are known to modify the mechanical properties of mature biofilms grown in vitro^30,45,46^. Five *P. aeruginosa* strains with genetically-modified patterns of polysaccharide production were used. All are based on the widely used laboratory strain PAO1 (Table 3)^47–51^. All strains used in this study constitutively express green fluorescent protein (GFP).

**Table 3:** PAO1 strains used.

Strain name	Description
WT	Wild Type – Produces primarily Psl and Pel
Δ*pel*	Produces no Pel
Δ*psl*	Produces no Psl
Δ*mucA*	Over-produces alginate
Δ*pel*Δ*psl*	Produces no Pel & no Psl

Polysaccharide production are based on in vitro observations.

### Fluorescent particles

For conducting particle-tracking passive microrheology experiments, 1.0 µm, carboxylate-modified, red fluorescent, latex particles (Invitrogen, Catalog Number #F88414) were used as probes. These particle’s size and surface coatings were chosen to allow the particles to embed into the biofilm matrix as it developed but satisfy typical microrheology requirements of being larger than and non-interacting with surrounding EPS, which has been demonstrated for these particles in previous experimental work^30,52^. To clean particles, they underwent several rounds of centrifuging, followed by removal of supernatant, and then suspension in fresh deionized (DI) water. A final particle solution was made with a concentration of 2 × 10^6^ particles/ mL, which we have found to provide sufficient particle numbers for tracking without creating numbers too large to make analysis difficult^25^.

### Bacterial culturing

An inoculating loop was used to place a small amount of frozen bacterial stock (stored at −80 °C) into 10 mL of freshly prepared, sterile LB medium (Luria-Bertani, from LB powder, Fisher Scientific, Catalog# BP1426-2) in a 100 mL, sterile, baffled, Erlenmeyer flask. The resulting culture was incubated for 16 h at 37 °C with shaking at 200 rpm.

For ex vivo studies, subcultures were prepared by diluting overnight cultures in fresh LB medium (1:100) and incubating for 3 h at 37 °C with shaking. The resulting subcultures were adjusted to an optical density of 0.4 at 600 nm (Thermo Scientific GENESYS 20 Spectrophotometer) and serially diluted (1:10) in phosphate-buffered saline (PBS) to 10^5^ colony forming units (CFU) per mL, which was verified by plating a diluted suspension on pre- LB agar plates using premixed LB agar (Fischer, BP9724) and counting the colonies that grew overnight.

For in-vitro studies, initial cultures were diluted to an optical density of 0.6 at 600 nm. 1 mL of this bacterial culture was centrifuged (10,000XG for 5 min) then washed and resuspended in fresh LB broth. The optical density of 0.6 at 600 nm (OD600) was reconfirmed by spectrophotometer after resuspension.

### Mouse chronic wound model biofilm microrheology (ex vivo)

All mouse procedures were carried out under protocol (#07044), approved by the Texas Tech University Health Science Center Institutional Animal Care and Use Committee in strict accordance with established guidelines at Texas Tech University, following recommendations in the Guide for the Care and Use of Laboratory Animals of the National Institute of Health as well as local, state, and federal laws.

**Fig. 4:**
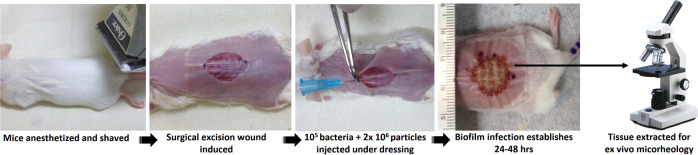
Schematic images of ex vivo mouse wound model particle tracking passive microrheology experimental method (Courtesy: Derek Fleming, Ph.D.; Rumbaugh lab, TTUHSC). All photographs were taken by authors.

The mouse chronic wound model has been used in previous studies^9,21,23,37,53–56^. Mice were anesthetized, and a dorsal, 1.5 × 1.5 cm excisional skin wound to the level of the panniculus muscle was administered. Wounds were covered with transparent, semipermeable polyurethane dressings (OPSITE dressings). 1.0 mL of fluorescent particle solution was spun down by centrifugation, the supernatant was removed, and particles were resuspended in 100 µL of bacterial suspension. The entire 100 µL suspension was injected under the dressings onto the wound beds. The infections were allowed to establish for 24 or 48 h, after which the animals were euthanized, and a thin section of the wound bed was harvested with a scalpel, placed on a microscope slide, flooded with 20 µL of PBS with antifade (Molecular Probes), and covered with a glass coverslip raised on spacers (Grace Bio-labs, SecureSeal imaging spacers) (Fig. 4).

**Fig. 5:**
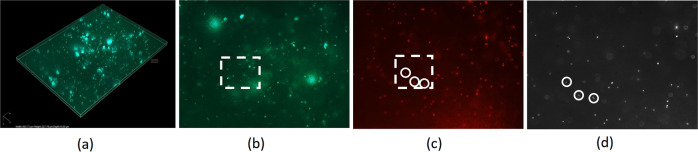
Representative microscopy data. **a** the 3D projection from confocal imaging z-stack of GFP expressing bacteria, **b** bottom layer of a z-stack of GFP expressing bacteria, **c** same field of view imaging red fluorescence to see embedded tracer particles, **d** corresponding image from the high-speed camera. Rectangles show the same field of view shown in (**d**) for FITC (green) and TRITC (red) channels in confocal mode. Circles show the same locations imaged in confocal mode (**c**) and the high-speed camera (**d**). All photographs were taken by authors.

Static Z-stacks of the freshly-harvested ex vivo wound bed biofilms were taken by manually adjusting focus with a Nikon eclipse 80i epifluorescence microscope using a 40x oil-immersion objective with a confocal A1 system and a Nikon Digital Sight Ds-Fi1 camera. Using the confocal scan, 5-layer z-stacks with 2 µm of space between each layer were taken of the infected tissue using green fluorescence to see GFP expressing *P. aeruginosa*. Figure 5a shows a representative stack from a single ex vivo technical replicate. From the bottom layer of the stack, we identified regions of high bacterial density, which correlate to areas of biofilm formation (Fig. 5b). At the same height as the bottom layer of stack, a single epifluorescence image of the red fluorescent beads was taken to identify particles located within identified biofilm (Fig. 5c).

Imaging for microrheology requires both a high frame capture rate and high spatial resolution, which the confocal microscopy camera system does not provide. Therefore, the light path was switched to an alternate camera port with a monochromatic high-speed camera (IL5, Fastec). At the same x, y-locations, and z height of the bottom layer of the 5-layer GFP Z-stacks, this camera took 1 min videos (at 50 frames/second) using epifluorescence imaging of the red fluorescent beads embedded in the ex vivo biofilms. In post processing, we identified the particles from the red fluorescence image that corresponded to areas of high bacterial density in the monochromatic image and used these for particle tracking analysis (Fig. 5d). Z-stacks and video were taken at four locations per sample, at locations where both a high density of particles and bacterial biofilm were observed. Total numbers of particles taken for each experiment are provided in Table 2 as well as number of biological and technical replicates.

### Microchannel biofilm microrheology

**Fig. 6:**
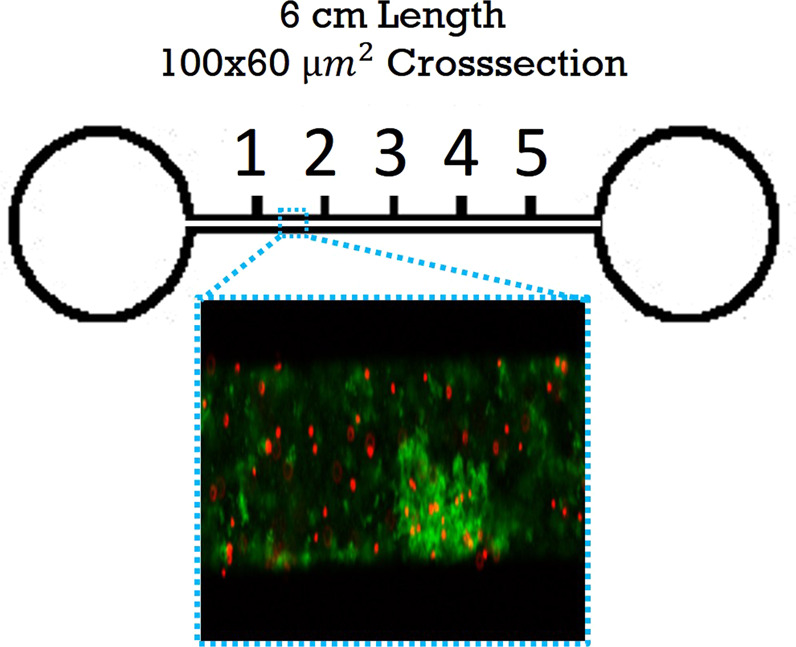
Microchannel schematic and example of biofilm with embedded particles. Locations (1,2,3…) are 1 cm apart. All photographs were taken by authors.

Microfluidic channels for in vitro biofilm growth (Fig. 6) were fabricated using standard soft lithography^57^. A channel mold was made using SU-8 negative photoresist (Su-8 2000, Microchem) spun onto a silicon wafer using the manufacturers guidelines to create a 60 micron thick layer. The Su-8 was patterned by placing a high resolution transparency mask (CAD/ART Services Inc) printed emulsion side down directly onto the Su-8 and exposing it with a UV flood exposure (Dymax, 2000-EC series) using a 380 nm filter. After cleaning and washing the mold as manufacturers instruction, polydimethylsiloxane (Sylgard 184, Dow Corning) with crosslinker was poured directly onto the molds and then degassed in a vacuum chamber overnight. For all experiments, the ratio of crosslinker to base was the manufacturer recommended 1:10. Crosslinker density affects surface properties of the channel and adhesion of cells^58^, but the effects on biofilm viscoelasticity are not established. However, these channels are typical of previously published work^25^. The polydimethylsiloxane channels were removed from the mold and access ports were punched with a 0.75 mm hole punch. The channel bottoms, made by spinning polydimethylsiloxane onto a glass slide, were bonded to channels using air plasma (Plasma Cleaner, Harrick Plasma), left overnight in at 80 °C oven, and stored at 20 °C until use.

The biofilms were grown and microrheology was conducted in a manner similar to previous work^25^. In brief, 50 µL of Wound Like Media^59^ (50% vol. Bovine Plasma (Fisher, Cat# 50-643-121), 45% vol. Bolton Broth (Fisher, Cat# OXCM0983B), and 5% vol freeze-thaw laked horse blood (VWR Cat# 10052-640)) was combined with 1 µL of particle suspension and 1 µL of the diluted bacterial culture. This mixture was injected into microchannels until they were full, and then inlets and outlets were sealed. Channels were placed in a static incubator at 37 °C. After 24 and 48 h, the microfluidic device was removed from the incubator and microrheology data was collected using a high speed camera (IL5, Fastec) connected to a Nikon Eclipse TS 100-F (Nikon Instruments) working in epi-fluorescence. Images were recorded at 50 fps with a 20x objective, for 4 distinct biofilms in each channel. Biofilms were identified using GFP fluorescence (Fig. 6). Data from all in vitro microchannel experiments were previously published^25^.

### Particle tracking microrheology and mean square displacement analysis

To probe the mechanical properties of biofilms, there are many tools available. However, most tools measure only the bulk response, damage the biofilm microstructure, and have high inter-experiment variation due to their protocols^60^. Particle-tracking microrheology can measure the spatio-temporal heterogeneity of mechanical properties without gross perturbation of biofilm microstructure^61^, and hence was chosen for this study.

Biofilms were seeded with probe particles as described above. Ambient thermal energy drives the motion of probe particles, thereby creating a local stress in the biofilm. Each particle’s displacement reflects strain arising from this stress. Individual particles are tracked, and both individual and ensemble mean square displacement (MSD) from particle trajectories are computed. The MSDs are analyzed using the Generalized Stokes-Einstein relationship^24^. The slope of the MSD-*versus*-lag time curve, $\alpha = \frac{{d\left( {In\left\langle {r^2\left( t \right)} \right\rangle } \right.}}{{d\left( {In\left( t \right)} \right)}}$, where *r* is position and *t* is lag time, ranges from 0 to 1 and characterizes the relative viscoelasticity of the material in which probe particles are embedded; *α* = 1 represents purely viscous diffusion whereas *α* = 0 represents an elastic solid^62^. Additionally, creep compliance can be calculated as a function of lag time, ${{{\mathrm{J}}}}\left( {{{\mathrm{t}}}} \right) = \frac{{3\pi {\it{a}}}}{{2k_BT}}\left\langle {r^2\left( t \right)} \right\rangle$, where *a* is the probe particle radius, *T* is the ambient temperature (298 K), and *k*_*B*_ is the Boltzmann constant (1.38 × 10^−23 ^J/K).

Particle locations and tracks from image sequences were found using ImageJ (Fiji installation) with the plugin TrackMate^63,64^, which locates particle centroids using a Laplacian of Gaussian filter, allowing subpixel localization. Generation of 2D trajectory tracks from particle locations is done using a simple linear assignment algorithm. Particle tracking data is converted to MSD vs.lag time curves using the MATLAB routine msdanalyzer^65^. Although lag times existed out to 10 s, we only produce results up to 1 s due to lower statistical significance at large lag times. Linear fits to the lowest 10% of lag times on each MSD curve^66^ were made to find the slope for in vitro data. Ex vivo data had fewer number of tracks/samples for every experiment (see Table 2), which makes each ensemble point representative of less data in comparison to in vitro data at similar lag times. We, therefore, chose to fit ex vivo data to lag times up to 1 s, which is not uncommon in microrheology with biofilms^36^.

### Statistical analysis

For the ex vivo wound bed samples, a single biological trial (i.e., 1 mouse) was done for each bacterial strain at 24 and 48 h after inoculation into the wound, resulting in two mice per bacterial strain. Multiple particles in each trial were characterized (Table 4). Due to the channel design used for in vitro experiments (Fig. 6), it was possible to index locations and gather data within the same biofilm after 24 and 48 h of biofilm growth. This process was repeated in three microchannels, resulting in three biological replicates and four technical replicates for in vitro experiments. The number of particle tracks for each trial varies, and the overall number is reported in Table 2.

**Table 4:** Number of particle tracks per bacterial strain for each set of experiments.

	Ex vivo					In vitro				
	WT	Δ*mucA*	Δ*pel*	Δ*psl*	Δ*pel*Δ*psl*	WT	Δ*mucA*	Δ*pel*	Δ*psl*	Δ*pel*Δ*psl*
24 h	69	43	77	92	154	301	396	235	177	1309
48 h	131	143	490	38	140	1233	629	187	439	1588

In vitro data reproduced from ref. ^25^ with permission from the Royal Society of Chemistry.

When examining *α* or *J*, typical analysis uses ensemble averages of values are used. Such ensemble averages, provide results that represent the average response of the biofilms. It is well established that particle position/depth within a biofilms, cell density, or biofilm thickness can create distinct microenvironments that affect rheological properties^36^. Although we do not specifically probe this, we do look at distributions of individual particles α or *J* using Box-whisker plot with statistical comparison. These plots show distribution of individual particles, which reflect the variability that naturally occurs in cell density, thickness, and depth.

At 24 and 48 h, the statistical significance of differences between the median *α* or *J* of each strain and the appropriate control was calculated. Ex vivo WT was the control for ex vivo measurements, and in vitro WT was the control for in vitro measurements. The null hypothesis was that a strains’ median value of α or *J* was equal to the median value of the control strain. Comparison of all strains to their WT control was done using a non-parametric Kruskal–Wallis one-way ANOVA method with Dunn’s multiple comparisons among tested groups^67^.

The statistical significance of differences between each bacterial strains’ ex vivo and in vitro results were also calculated. The null hypothesis was that the in vitro value and the ex vivo value for the same bacterial strain were equal. Ex vivo and in vitro results were compared using a Mann–Whitney test.

The significance levels were set at *p*-value > 0.05 not significant, 0.05 > *p*-value ≥ 0.005(*), 0.005 > *p*-value ≥ 0.0001 (**), 0.0001 > *p*-value (***).

### Reporting summary

Further information on research design is available in the Nature Research Reporting Summary linked to this article.

## Supplementary information

Reporting Summary

## Data Availability

The datasets generated during and/or analyzed during the current study are available from the corresponding author on reasonable request.

## Code availability

All code used in analysis of data is comes from free, publicly available sources.
